# Comparative Study of FSW, MIG, and TIG Welding of AA5083-H111 Based on the Evaluation of Welded Joints and Economic Aspect

**DOI:** 10.3390/ma16145124

**Published:** 2023-07-20

**Authors:** Mohamed I. A. Habba, Naser A. Alsaleh, Takwa E. Badran, Mohamed M. El-Sayed Seleman, Sabbah Ataya, Ahmed E. El-Nikhaily, Akrum Abdul-Latif, Mohamed M. Z. Ahmed

**Affiliations:** 1 Mechanical Department, Faculty of Technology and Education, Suez University, Suez 43518, Egypt; mohamed.atia@suezuniv.edu.eg (M.I.A.H.); takwabadran6@gmail.com (T.E.B.); ahmed.eassa@ind.suezuni.edu.eg (A.E.E.-N.); 2 Department of Mechanical Engineering, College of Engineering, Imam Mohammad Ibn Saud Islamic University, Riyadh 11432, Saudi Arabia; naalsaleh@imamu.edu.sa (N.A.A.); smataya@imamu.edu.sa (S.A.); 3 Department of Metallurgical and Materials Engineering, Faculty of Petroleum and Mining Engineering, Suez University, Suez 43512, Egypt; mohamed.elnagar@suezuniv.edu.eg; 4 IUT de Tremblay-en-France, Université Paris 8, 93290 Tremblay-en-France, France; akrum.abdul-latif@univ-paris8.fr; 5 Mechanical Engineering Department, College of Engineering at Al Kharj, Prince Sattam bin Abdulaziz University, Al Kharj 11942, Saudi Arabia

**Keywords:** AA5083-H11, friction stir welding, tungsten inert gas (TIG), metal inert gas (MIG), radiographic inspection, mechanical properties, fracture surface, welding time and cost

## Abstract

Selecting an economically suitable welding technique and optimizing welding parameters to obtain high joint quality is considered a challenge for expanding the 5xxx aluminum alloy series in various industrial applications. This work aims to investigate the effect of applying different welding techniques, tungsten inert gas (TIG) and metal inert gas (MIG), as fusion welding processes compared to friction stir welding (FSW), a solid-state joining process, on the joint performance of the produced 5 mm thick similar AA5083-H111 butt weldments at different welding conditions. Different methods were used to evaluate the quality of the produced joints, including visual inspection, radiographic testing (RT), and macrostructure evaluation, in addition to hardness and tensile tests. The fracture surface of the tensile-failed specimens was also investigated using a scanning electron microscope (SEM). Furthermore, the current study ended with an economic analysis of the welding techniques used. The results showed that, for the friction stir-welded joints, the radiographic films revealed defect-free joints at the two applied travel speeds of 100 mm/min and 400 mm/min and a constant tool rotating speed of 400 rpm. In addition, only one joint was welded by MIG at a welding current of 130 Amp, with a 19 L/min flow rate of pure argon. In contrast, the radiographic films showed internal defects such as lack of fusion (LOF), lack of penetration (LOP), and porosity (P) for the two joints welded by TIG and one joint welded by MIG. The hardness of the welded joints was enhanced over the AA5083-H111 base material (BM) by 24–29, 31–35, and 46–50% for the MIG, TIG, and FSW joints, respectively. The maximum ultimate tensile strength was obtained for the FSW joint welded at a 400 mm/min travel speed. Adopting FSW in shipbuilding applications can further produce the AA5083-H11 joints with higher quality and efficiency than fusion welding techniques such as MIG and TIG processes. In addition, time and cost comparisons between TIG, MIG, and FSW were performed for five-millimeter-thick and one-meter-long AA5083-H111.

## 1. Introduction

Non-heat-treatable aluminum alloys are used in many critical industrial applications due to their high strength-to-weight ratio, good machinability, and high corrosion resistance [1,2,3]. A good representative of these materials within the 5xxx (Al-Mg alloy) series alloys is AA5083 aluminum alloy, which is used in applications requiring high specific strength and high corrosion resistance, including shipbuilding, rail carriages, and vehicle bodies [4,5]. Because there is a real need in such industries to make joints of this alloy, developing an economically suitable welding method and optimizing the welding parameters is considered a challenge for expanding its industrial applications. Fusion welding processes are the most common technique for joining metal alloys. Two main fusion welding processes are used in the joining process [6,7]. The first is the tungsten inert gas (TIG) welding process, which involves the formation of an electric arc between the non-consumable tungsten electrode and the welding plates. This arc should provide sufficient energy to melt the welding plates in the weld zone and the filler metal [8,9,10]. The second process is the metal inert gas (MIG) welding process, which generates a constant electric arc between an automatically fed consumable electrode and a joint plate in the weld zone with an inert gas shield [10,11,12]. Compared to the TIG technique, the MIG technique is faster and can be used for joining thick sections. In addition, the MIG-welded joints are generally stronger than the TIG joints due to their high penetration [6,7].

Friction stir welding (FSW) is a solid-state welding technique. This method involves an advancing, non-consumable rotating tool used to join two facing surfaces of various alloys without needing filling materials [13,14,15]. The frictional heating generated during the FSW process is related to the welding variables involved, including tool design [16,17], rotating speed [18], welding speed [19], and tilt angle [20,21]. This heat is generated by the direct contact between the rotating tool shoulder and the surface of the target material for welding, plus the action of the rotating pin to stir and mix the two contact surfaces of the welding samples. When adjusting the FSW parameters, the generated heat input is sufficient to bring the material under the welding procedure to the plasticity stage (without melting), which facilitates the joining process [22,23,24]. Recent works have focused extensively on the FSW welding of similar and dissimilar aluminum alloys, as they are a good alternative to heavy materials in modern transportation industries [25,26,27]. Based on the published data, several works aimed to explore only the impact of certain parameters on the joint quality of the FSW of AA5083-H111 [28,29,30]. However, comparative studies between this solid-state technique and the fusion welding techniques are few, especially related to the AA5083-H111 welding joints [1,31,32]. Table 1 summarizes the output of previous works that compare the FSW process and fusion welding techniques in terms of MIG and TIG.

Furthermore, there is still a need in the industrial sector for more comparative studies between the different types of welding techniques to produce defect-free joints with acceptable mechanical properties for the AA5083-H111 aluminum alloy, keeping in mind cost-effectiveness.

Consequently, the aim of the present study is to compare the impact of applying FSW technology with MIG and TIG techniques, which are intensively used fusion welding techniques to achieve 5 mm thick, similar AA5083-H111 butt joints. Two joints were welded at different welding conditions for each welding technique. For the TIG and MIG processes, the produced AA5083-H111 butt joints were welded at different heat inputs. Additionally, for the FSW process, the two different welding speeds of 100 and 400 mm/min were applied using a constant tool rotation speed of 400 rpm. The effects of welding techniques and their parameters on the quality of welded joints were studied via visual examination. non-destructive testing, microstructure, hardness, and tensile properties. The tensile-failed specimens were also investigated using a scanning electron microscope (SEM). Finally, the study ended with an economic analysis.

## 2. Materials and Methods

### 2.1. Material

AA5083-H111 aluminum alloy rolled sheets were welded in butt joint configuration utilizing TIG, MIG, and FSW welding techniques. The AA5083-H111 sheets with 5 mm thickness (T) were cut into 200 mm length (L) × 100 mm width (W) plates for welding using the different welding techniques suggested. Before the welding processes (MIG, TIG, and FSW), all edges of plates were carefully cleaned mechanically and chemically to eliminate any source of contamination, such as rust, dust, oil, and moisture, that may penetrate through the weld zone, causing a weld defect. The welding paths are parallel to the rolling direction of the AA5081-H111 plates. Table 2 lists the nominal composition of the AA5083-H11 base material according to the supplier, OCEANDRO Shipyard Company for Ship Building and Repair, Adabiya, Suez, Egypt.

### 2.2. Fusion Welding of AA5083-H111

The AA5083-H111 plates were fusion welded using two different techniques: TIG and MIG welding processes. The plates were machined to obtain a V-groove with an angle of 70°, a 3 mm root gap, and a 2 mm root face for the TIG and MIG welding processes. In the MIG (Lorch S5, Auenwald, Germany) welding process, the prepared plates were welded with ER5356 (ESAB, OK Autrod 5356, Gothenburg, Sweden) with a 1.2 mm diameter. According to the supplier (ESAB AB, Gothenburg, Sweden), the typical composition of the electrode wire is listed in Table 3. In the TIG (Lorch T300, Auenwald, Germany) welding process, the AA5083-H111 joint was welded using commercial R5356 (ESAB, OK TIGROD 5356) filler wire, whose diameter was 2.4 mm and length was 100 mm. The chemical composition of the filler wire, according to the supplier (ESAB AB, Gothenburg, Sweden), is presented in Table 3. The fusion welding parameters were selected based on our experience and literature [17,37,38]. The applied MIG and TIG welding parameters are presented in Table 4.

### 2.3. FSW of AA5083-H111

**Figure 1:**
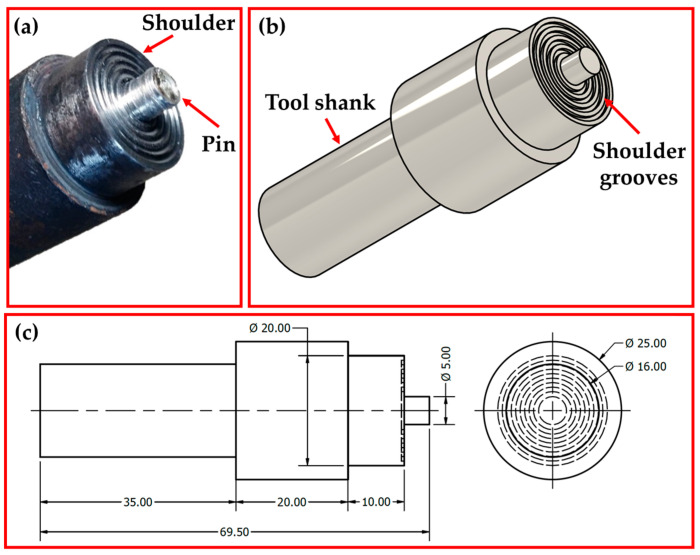
(**a**) photograph, (**b**) 3D sketch, and (**c**) dimensions (in mm) of the FSW tool.

FSW of AA58383-H111 was performed using an Egyptian friction stir welding machine (Model-EG-FSW-M1, Suez University, Suez, Egypt). The FSW tool was designed with a smooth cylindrical pin geometry and a concave (5°) shoulder with grooves, as shown in Figure 1. The shoulder diameter, pin length, and pin diameter were chosen as 20 mm, 5 mm, and 4.5 mm, respectively. The FSW tool was fabricated from W302 tool steel and heat treated to achieve a hardness of 62 HRC. Based on preliminary FSW experimental trials and our experience, the applied FSW parameters to achieve similar AA5083-H111 butt joints were a constant tool rotation speed of 400 rpm and two different travel speeds of 100 (Sample Code: F1) and 400 mm/min (Sample Code: F2) at axial downward forces of 14.61 and 12.35 KN, respectively. The tool tilt angle and plunge depth during the FSW process were 3° and 4.8 mm, respectively.

### 2.4. Characterization of AA5083-H111 Welds

**Figure 2:**
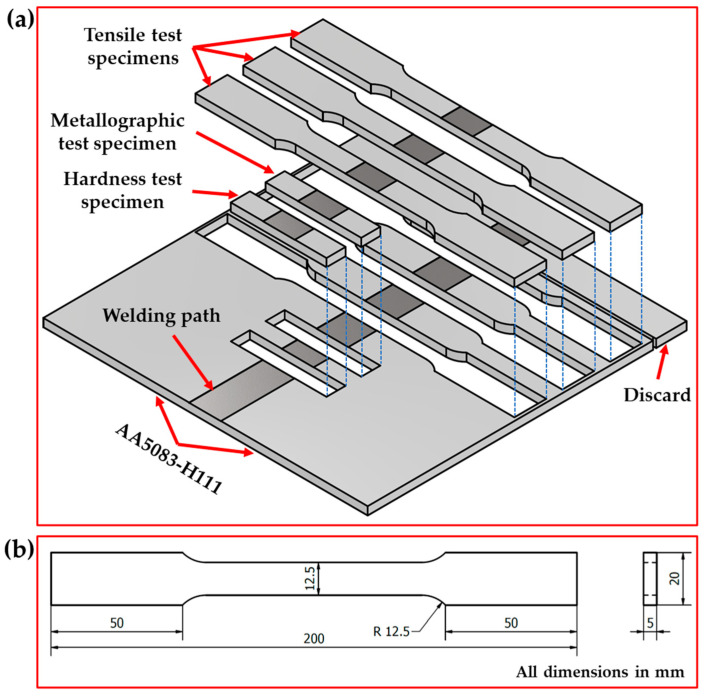
The illustration sketch shows (**a**) the location of the test samples cut from the weld joints for different tests and (**b**) the dimensions of the tensile test specimen.

**Figure 3:**
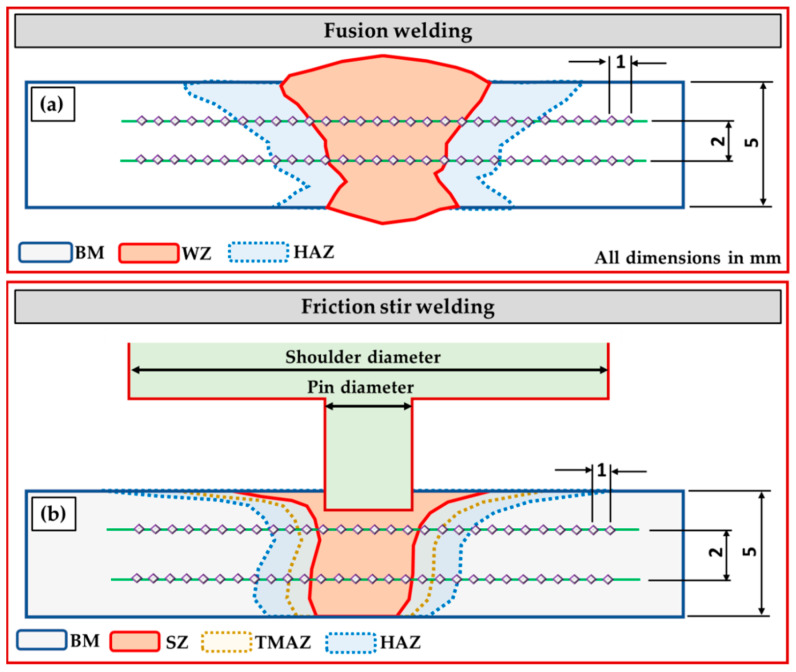
Schematic drawing of hardness measurements on the cross-sections of (**a**) the fusion welding joints and (**b**) the FSW joints.

After welding processes, the welded samples were evaluated via visual inspection, radiographic inspection, metallographic test, Vickers hardness test, and tensile test to compare the effect of welding processes on the quality of joining 5 mm AA5083-H111 in similar butt welds used in shipbuilding applications. Figure 2a illustrates the cut test samples for all the applied welding techniques from the welding paths. The visual and radiographic inspections were carried out at PETROJET Company (the Petroleum Projects and Technical Consultations Company), Suez, Egypt. The radiographic inspection test was carried out according to ASME V and ASME VIII on the welded joints using a Gamma-ray camera (880 MAN-027, Glenwood, NSW, Australia) with an Iridium-192 source using D7 radiographic films. For macrostructure, hardness, and tensile tests, the AA5083-H111-welded joints were cut using different techniques perpendicular to the welding path direction, as shown in Figure 2a. Figure 2b shows the tensile test sample dimensions according to ASTM-E8 04 [39]. The cross-sections of the welded specimens for macrostructure investigation were ground with emery paper up to 2500 and polished using a 0.5 µm Al_2_O_3_ paste, followed by an etching process using Keller’s reagent. The Vickers hardness test was performed along a prepared linear direction perpendicular to the weld path at the mid-thickness of the cross-section of the AA5083-H111-welded joints. The hardness measurements were carried out using a Vickers hardness tester (HWDV-75, TTS Unlimited, Osaka, Japan) with a 200 g applied load and a 15 s dwell time with 1 mm spacing between each measurement, as shown in Figure 3a,b. The tensile test of the welded joints was evaluated using a universal testing machine (WDW-300D Testing Machine, Guangdong, China). The tensile specimens’ fracture surfaces were investigated using a scanning electron microscope (SEM-Quanta FEG 250—FEI Company, Hillsboro, OR, USA).

## 3. Results and Discussion

### 3.1. Visual Inspections

**Figure 4:**
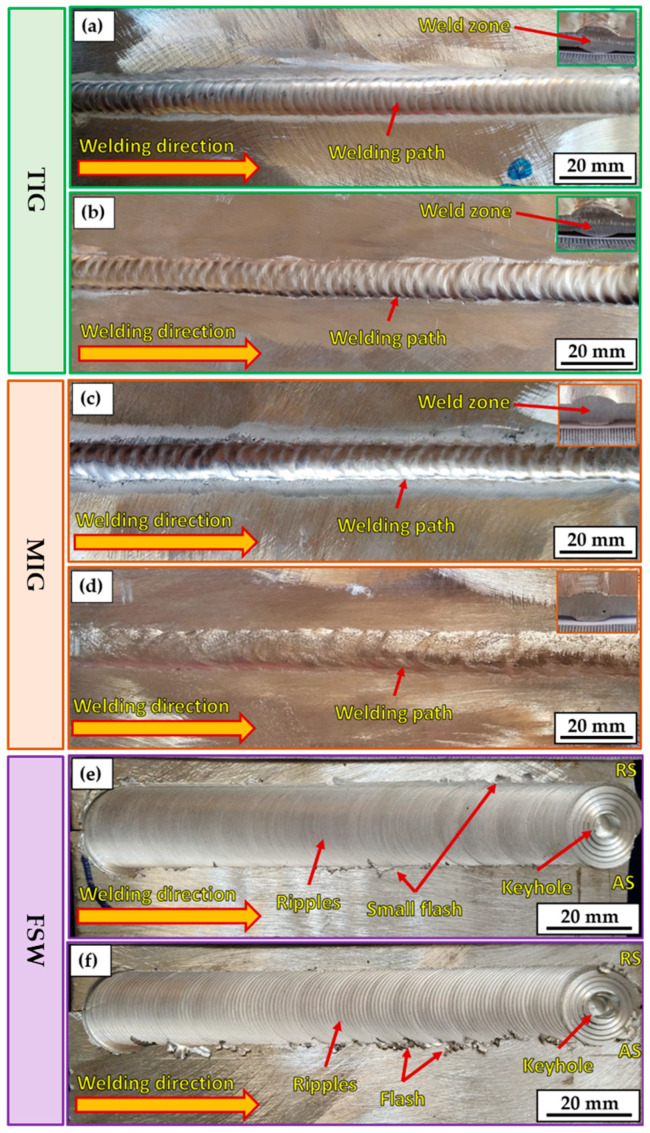
Top-view photo images of the welded joints of the TIG (**a**,**b**), MIG (**c**,**d**), and FSW (**e**,**f**).

The most popular way to determine the surface quality of a weld is via a visual inspection of the weldments. This kind of inspection, when carried out correctly, may often be a very efficient way to maintain acceptable welding quality and avoid welding problems. Figure 4 depicts the top view of AA5083-H111-welded joints using different welding processes: TIG (Figure 4a,b), MIG (Figure 4c,d), and FSW (Figure 4e,f). It can be remarked that for all the welded joints, the different welding processes successfully welded 5 mm thick AA5083-H111 in butt joints. Moreover, it can be considered acceptable without any defects on the surface of the welded joints. Table 5 summarizes the result of the visual examination of the AA5083-H111 butt joints welded using the three different welding techniques. It can be concluded that both sides (upper and lower surfaces) of all welded joints are acceptable. However, there are some minor observations. The high joint reinforcement of the TIG and MIG joints ranged from 1.5 to 3 mm, as listed in Table 5.

In contrast, the thickness of the FSW-welded joints is reduced from 5 to 4.8 mm. Additionally, it can be mentioned that the width of the welding pass is approximately 12 mm for both fusion welding processes. In the case of FSW, the welding pass varied from top to bottom across the joint thickness due to the FSW tool dimensions of the shoulder (20 mm) and pin (5 mm). A small flash was observed on the two sides of the welding path for FSW joints (Figure 4e,f). The excessive welding flash is considered a material loss and commonly appears due to excessive heat input during the stirring action of the FSW tool, where plasticized material under the shoulder area extrudes on both sides of the weld path in the form of a welding flash [40]. Furthermore, it was reported [36] that the flash formed on the advanced side (AS) is higher than the retreating side (RS) due to the stirring mechanism during the FSW process, in which the material in the stir zone flows the plasticized material from AS to RS [41]. Thus, FSW welding variables should be considered to control the formation of an excessive welding flash, including tool design, plunge depth, tilt angle, and introduced heat input [42,43]. Hence, the FSW machine parameters (400 rpm rotation speed, 100 and 400 mm/min travel speeds, 4.8 mm tool plunge depth, and 3° tilt angle) and the FSW tool design should be chosen carefully to reduce flash forming during the welding process of AA5083-H1111.

Particularly in the FSW process, the keyhole forms at the end of the weld path (Figure 4d,f), resulting from the extraction of a non-consumable tool pin after the end of the welding process. However, many techniques have been suggested to fill the keyhole in FSW welds [44,45]. The FSW-welded joints had a clear and defect-free ripple generation process. The contact between the tool’s shoulder and the top surface of the joint plates during FSW produced the rippling space, which was about equal to the gap between the circle’s circumferences. The softened material’s solidification caused the ripple configuration to develop. The travel speed impacted the weld bead structure, observable as ripples. It can be mentioned that the distance between formed ripples increased with increasing the travel speed from 100 to 400 mm/min during the string action between the rotation tool and joint material during the FSW process. These findings agree with those reported in previous works [46,47].

### 3.2. Radiographic Inspection

**Figure 5:**
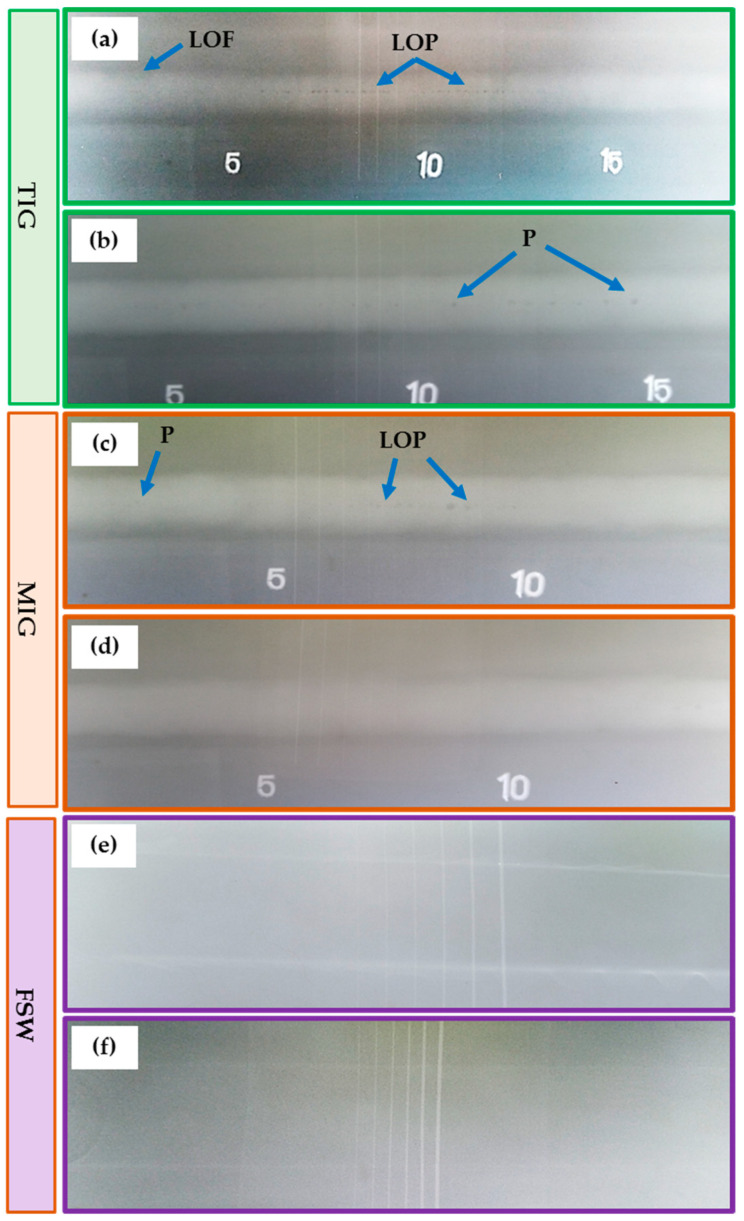
Radiograph films of the welded joints of (**a**) T1, (**b**) T2, (**c**) M1, (**d**) M2, (**e**) F1, and (**f**) F2 using different welding techniques to produce AA5083-H111 butt joints.

Radiographic testing (RT) detects internal defects in the internal structure of the welding path. Figure 5 shows the radiographic films of the TIG (Figure 5a,b), MIG (Figure 5c,d), and FSW (Figure 5e,f) weldments. Furthermore, Table 6 summarizes the RT results for the welded joints. For the TIG joints (T1 and T2), some internal defects appeared, such as lack of fusion (LOF), lack of penetration (LOP), and porosity (P), as shown in Figure 5a,b. Additionally, porosity and LOP defects are detected in the MIG M1 joint (Figure 5c).

Moreover, the RT revealed a sound joint for the M2, F1, and F2 weldments, as shown in Figure 5d–f, respectively. For the fusion based techniques, it can be mentioned that the welding porosity is caused by entrapped gases during the weld solidification. The LOF happens when the weld is not fused with the joint material. Furthermore, the LOP occurs when the weld seam is inadequate to fill the root of the joint. The LOF and LOP are caused by the low heat input from the high welding speed or the low current applied [48,49,50].

### 3.3. Macrostructure

**Figure 6:**
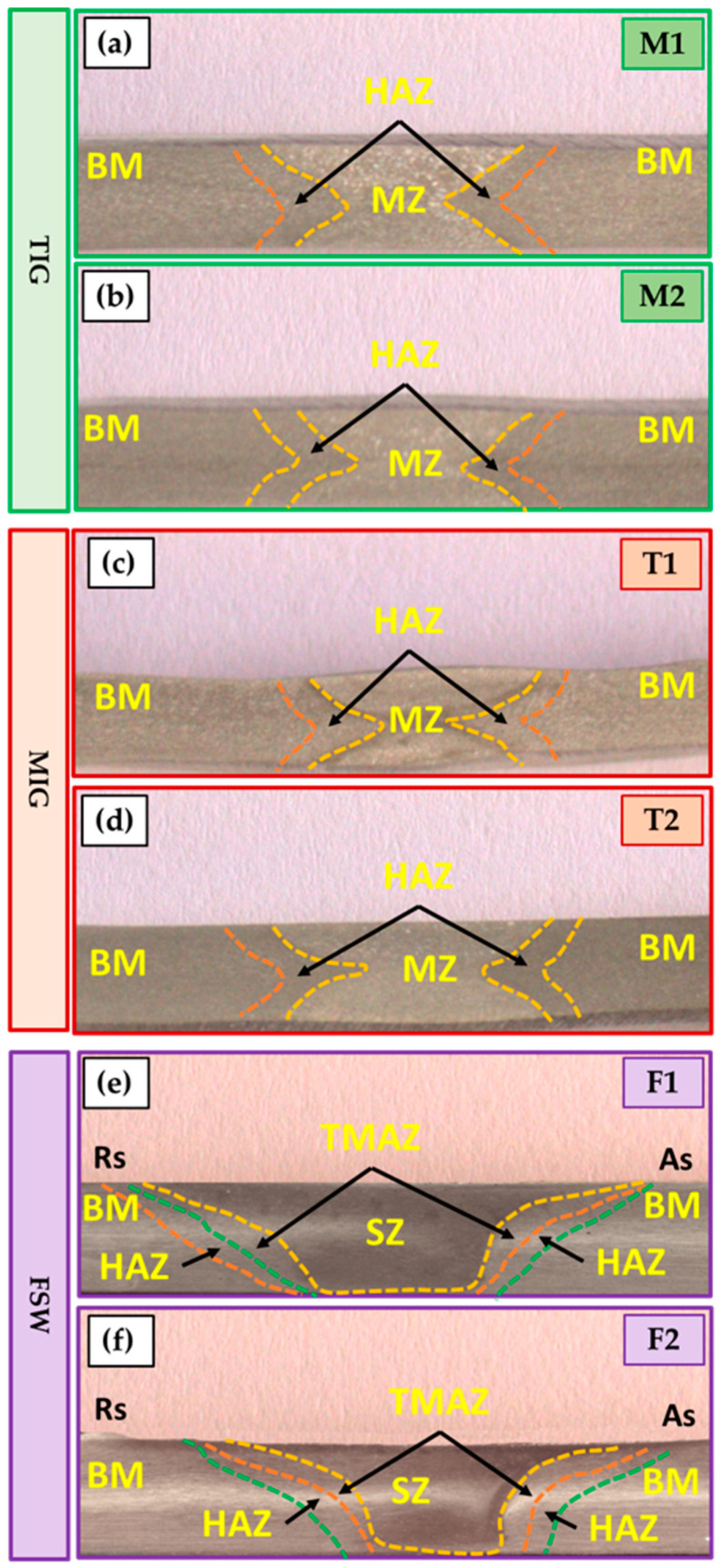
Macro-images of the AA5083 butt joints welded using different techniques of (**a**,**b**) TIG, (**c**,**d**) MIG, and (**e**,**f**) FSW.

Figure 6 depicts the macrostructure of the cross-section of the 5 mm thick AA5083-H111 butt joints welded using TIG (Figure 6a,b), MIG (Figure 6c,d), and FSW (Figure 6e,f) techniques. No defects such as tunnels or cracks were noted in all the investigated cross-sections of the welded joints using the three welding techniques. In addition, as expected, the welding features of WZ (weld zone) and HAZ (heat-affected zone) are typical for the fusion welding techniques (TIG and MIG). SZ (stir zone), TMAZ (thermomechanically affected zone), and HAZ are features of the FSW process. For the fusion welding, it is evident that the WZ showed a dumbbell shape in the two processes of TIG and MIG due to the welding procedure (Table 4) and joint design. Furthermore, no welding defects such as large pores and undercuts have been observed. For the FSW joints of the F1 and F2 joints (Figure 6e,f), there are also no defects, and the WZ gives a basin shape. The interface between the SZ and the TMAZ is very distinct on the AS. In addition, the RS is more dispersed owing to the alignment of the rotation direction and the transverse direction on the RS. The BM is an unaffected zone by the FSW process and exhibits the typical microstructures of the rolling sheets of aluminum alloy. The HAZ zone is influenced by the heat produced during the FSW process and is characterized by a variation in precipitate size, distribution, and grain growth compared to the BM [51,52,53]. The TMAZ is affected by plastic deformation (string action) and heat generation during the FSW process. The presence of relatively randomly oriented grains with respect to one another in this zone is its defining feature. Since the amounts of temperature and deformation experienced are insufficient to cause dynamic recrystallization, these grains are generated mainly by dynamic recovery [53,54]. Finally, a refined equiaxed grain structure can be seen in the SZ [43,53]. Since the heat generated and plastic deformation are sufficiently high, in contrast to the TMAZ, to induce this phenomenon, the new grain structure is formed by continuous dynamic recrystallization [53,55,56,57].

### 3.4. Mechanical Properties

#### 3.4.1. Hardness of the Produced Joints

**Figure 7:**
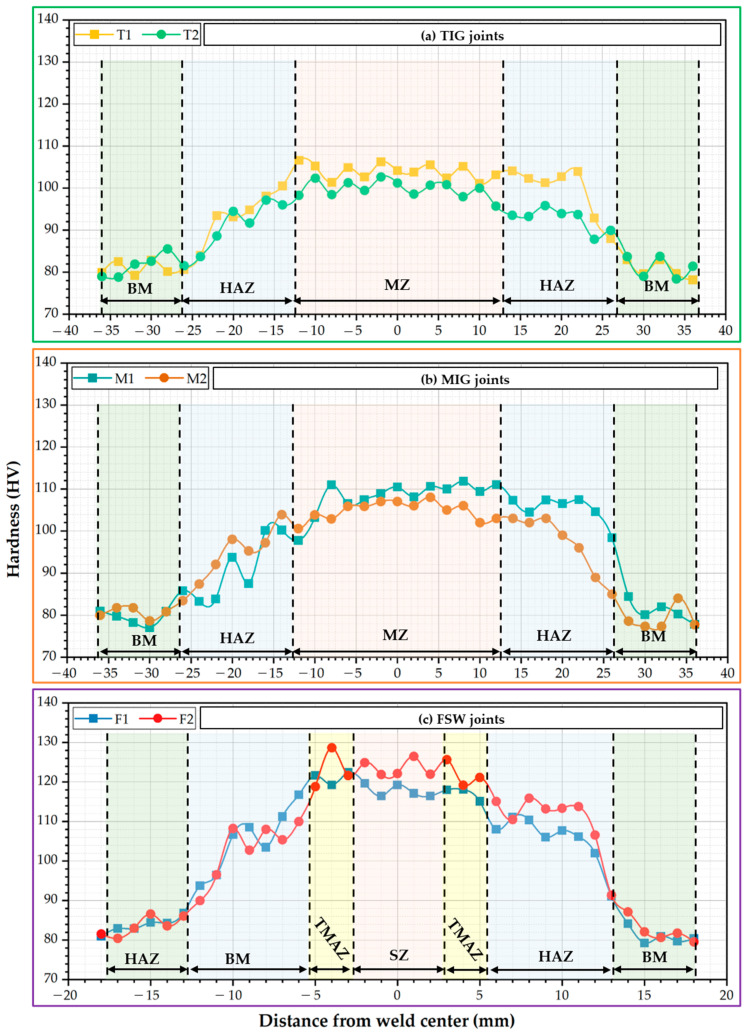
Vickers hardness results of 5 mm AA5083 MIG, TIG, and FSW butt joints.

Figure 7 presents the Vickers hardness profile of the cross-section of the welded joints: (a) TIG, (b) MIG, and (c) FSW. Generally, the hardness of the welded joints increases in the different zones of the welded joints, such as WZ and HAZ for the TIG and MIG joints and SZ, TMAZ, and HAZ for the FSW joints. While moving from the center weld, and all welded joints to BM, a decreasing tendency in the order of weld zone (WZ or SZ), TMAZ, HAZ, and unaffected BM was marked. For TIG weldments, the average hardness of WZ was 103 ± 3.3 HV for the T1 joints and 99 ± 2.4 HV for the T2 joints, with improvements of around 29% and 24%, respectively, compared to the hardness of AA5083-H111 BM (80 ± 2.1 HV), as plotted in Figure 7a. In the MIG weldments, the hardness of the WZ was 108 ± 4.2 HV for M1 and 105 ± 2.7 HV for M2 joints, with enhancements of around 35% and 31%, respectively, compared to BM, as given in Figure 7b.

Furthermore, the hardness of the HAZ of the welded joints in both fusion welding techniques slightly decreased by around 4% compared to the WZ hardness, as shown in Figure 7a,b. The TIG and MIG weldments were noted to have lower hardness values owing to the presence of coarse particles, cast microstructure, and the evaporation of magnesium elements caused by the influence of the high temperature during fusion welding [58]. However, the WZ displays a higher hardness than the BM, utilizing the ER5356 filler rod, which has 5% Mg, as listed in Table 2. Incorporating magnesium into aluminum alloys increases the material’s hardness [58]. Liyakat and Veeman [59] detected intermetallic phases such as Mg_2_Si and Al_3_Mg_2_ during the TIG welding of AA5052-H32 [59]. Thus, the hardness of TIG and MIG weldments increased due to the formation of intermetallic phases in the WZ compared to the BM. In addition, the decrease in hardness near the HAZ for fusion weldments is due to the loss of strain hardening effect and grain coarsening during fusion welding (High-temperature cycle). The coarsening of strengthening intermetallic particles reduces HAZ hardness [58].

On the other hand, the hardness measurements of the welded joints using the FSW process have the same trend as the fusion-welded joints. For the FSW joints, the average SZ hardness was 117 ± 1.1 HV for the F1 joints and 121 ± 1.3 HV for the F2 joints, with improvements of around 46% and 50%, respectively, compared to the BM, as shown in Figure 7c. Furthermore, the hardness of the FSW joints is higher than that of the fusion welding joints of TIG and MIG. During the FSW process, severe plastic deformation of the material was caused by string action in the SZ. This might change the weld bead microstructure to attain grain refinement in the SZ. Ahmed et al. [60] studied the effect of FSW travel speeds ranging from 50 to 200 mm/min on the microstructure features and mechanical properties of similar and dissimilar joints of AA5083 and AA7075 aluminum alloys while keeping tool rotation speed and tool tilt angle constant at 300 rpm and 3°, respectively. For the similar AA5083-H111 joints, they recorded high grain refining reaching 9 and 3 µm in the SZ for the FSW joints at 50 and 200 rpm, respectively, compared to 25 µm for the as-received AA5083-H111. They attributed this phenomenon to dynamic recrystallization. Because aluminum has a high stacking fault energy that leads to high rates of dynamic recovery, geometric dynamic recrystallization and continuous dynamic recrystallization are assumed to lead to newly refined grains forming during the hot plastic deformation. These formed fine grains positively improve the hardness values in the SZ compared to the BM, as noticed in Figure 7c. In addition, the hardness values increase with increasing welding speed from 100 to 400 mm/min due to the decrease in the generated heat input during the welding process.

#### 3.4.2. Tensile Properties

**Figure 8:**
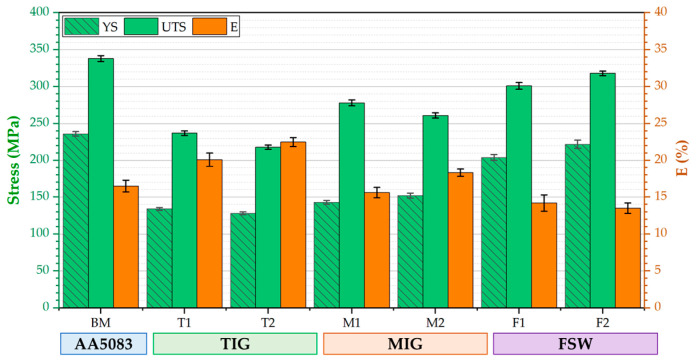
Ultimate tensile strength, yield strength, and elongation perecntage of the similar AA5083-H111 but joints welded using different techniques.

Tensile tests were performed on the AA5083-H111 BM and its welded joints using the TIG, MIG, and FSW techniques to determine tensile properties such as ultimate tensile strength (UTS), yield strength (YS), and elongation (E%). Figure 8 illustrates the tensile test results of the TIG, MIG, and FSW joints. It can be seen that the values of UTS, YS, and E% of the AA5083-H111 BM were recorded to be 338 MPa, 236 MPa, and 16.5%, respectively. The values of the UTS of the T1, T2, M1, M2, F1, and F2 tensile test specimens for the welded joints recorded 237, 218, 278, 261, 301, and 318 MPa, respectively. Furthermore, E% of tested specimens revealed 20.1, 22.5, 15.6, 18.3, 14.2, and 13.5% for the joints coded with T1, T2, M1, M2, F1, and F2, respectively. In addition, the YS was 236, 134, 128, 143, 152, 204, and 222 MPa for the welded joints of T1, T2, M1, M2, F1, and F2, respectively. For TIG and MIG joints, the fracture locations of tested specimens were located in the WZ due to the cast structure of the WZ (fusion welding process) and the coarse grain formed during the high temperature of the WZ [61]. Furthermore, the common fusion welding defects such as LOF, LOP, and P were observed (Figure 5) for the T1, T2, and M1 joints, which could be the motive for crack initiation during the testing of tensile specimens when failure is located in the WZ.

**Figure 9:**
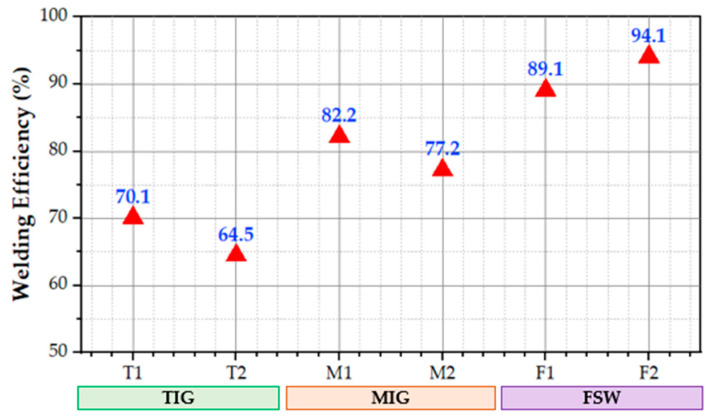
The welding efficiency of the welded joints using TIG, MIG, and FSW processes.

In contrast, the fracture location of the F2 (FSW joints) was located at the HAZ between the SZ and BM. Furthermore, the F1 joints failed at the SZ of the tested specimens. The higher UTS for each applied welding technique is T1, M1, and F2, with joint efficiency of 70, 83, and 89% compared to the UTS of the AA5083-H111 BM. The tensile properties of the fusion weldments (TIG and MIG) were unable to reach BM due to the effect of high heat input beside the WZ, inducing it to lose the strain hardening effect of the AA5083-H111 BM. During the FSW of the AA5083-H111, the low heat input during the welding process (solid state welding process) has a much lesser effect on HAZ compared to the fusion welding process. Additionally, the grain refinement and strain hardening occurring during the FSW process in the SZ [62,63,64] improve the FSW joints’ UTS more than the fusion welding joints. Congruently, the reduction in E% for FSW joints is expected (Figure 7). Figure 9 shows the joint efficiency of the TIG, MIG, and FSW joints. It can be mentioned that the maximum welding efficiency of TIG, MIG, and FSW joints is 70.1, 82.2, and 94.1%, respectively. These results indicate that the joints welded using the FSW technique have higher joint efficiency than the fusion welding techniques of TIG and MIG.

A material’s hardening capacity (*Hc*) may be considered a ratio of the UTS to the YS [65,66]. Kumar et al. [65] defined the Hc as a normalized parameter,

(1) $$Hc=\frac{UTS-YS}{YS}=\frac{UTS}{YS}-1\;$$

**Figure 10:**
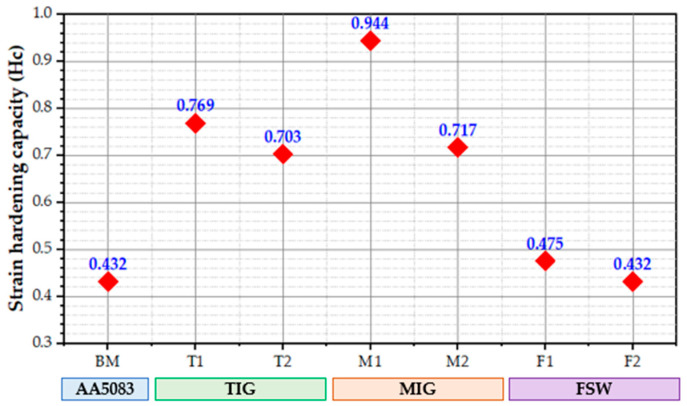
The hardening capacity of the AA5083-H111 BM and the TIG, MIG, and FSW joints.

Following Equation (1), the values of *Hc* of the welded joints using different welding techniques are displayed in Figure 10. According to Equation (1), the *Hc* is related to a *YS* of tested materials. *YS* increases when a material is strengthened, but *Hc* decreases because dislocation storage capacity decreases during plastic deformation. For instance, the *YS* of the T1 joint decreased by an average of 134 MPa, and *Hc* increased by 0.769 when the TIG process was applied to weld AA5083-H111. In contrast, the *YS* increased by an average of 222 MPa, and the corresponding value of *Hc* decreased by an average of 0.432 when the FSW was applied to weld AA5083-H111 using a 400 rpm rotation speed and a travel speed of 400 mm/min. It can be concluded that the welding techniques and their parameters almost have the same effect on the *Hc*.

**Figure 11:**
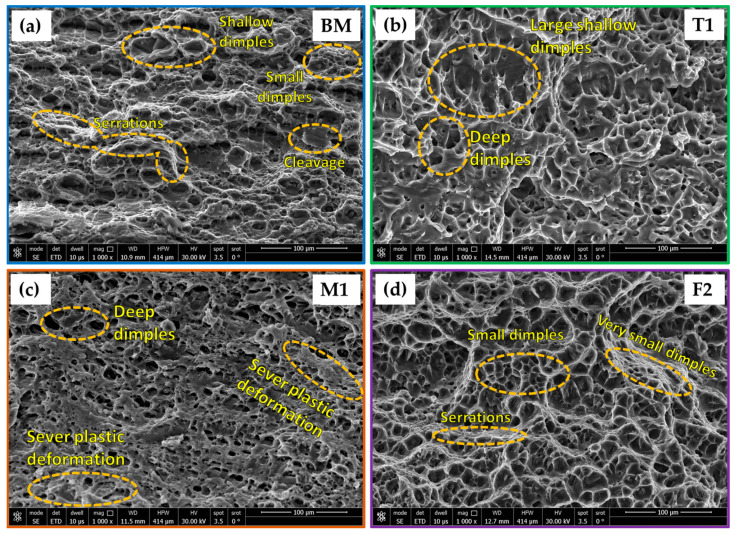
The fracture surface of the tested tensile specimens of the (**a**) AA5083-H11 BM and their welded joints coded with (**b**) T1, (**c**) M1, and (**d**) F2.

The SEM images of the fracture surface morphology of the AA5083-H11 BM (Figure 11a) and the welded joints coded with T1 (Figure 11b), M1 (Figure 11c), and F2 (Figure 11d) are given in Figure 11. The SEM of AA5083-H111 BM displayed different sizes of dimples with some areas of cleavage and serrations, indicating mixed fracture modes (ductile and brittle). An observable difference exists in the shape and size of the dimples and other fracture features concerning welding techniques [67,68]. For the dimples feature, coarse dimples were detected at the fractured surfaces of the T1 (TIG) and M1 (MIG) welded samples, and fine dimples were at the fractured surfaces of the F2 (FSW) joint. The FSW joints (F1 and F2) have revealed lower E% than TIG (T1 and T2) and MIG (M1 and M2) joints, which may be why FSW joints have higher UTS compared to those of TIG and MIG welds.

### 3.5. Economic Analysis

In this section, the economic comparisons between the selected TIG, MIG, and FSW welding techniques of 5 mm thick AA5083-H111 were analyzed according to time and cost items. The economic comparison was completed by welding a 1 m long, 5 mm thick AA5083-H111 sheet. The time and cost of preparing the sheets to a given width and length of joint plates will not be considered.

#### 3.5.1. Time of Preparation and Welding Processes

**Figure 12:**
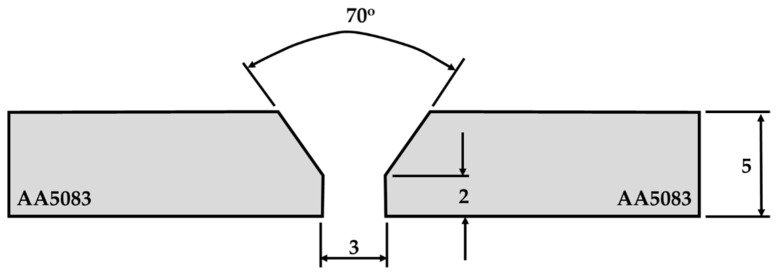
Sketch of preparation joints for TIG and MIG welding processes.

A remarkable problem in the fusion welding of aluminum alloys is forming the oxide layer, which is continuously formed on the aluminum surface during welding. The oxide layer must be removed from the initial material before fusion welding aluminum alloys. For the fusion welding processes (MIG and TIG), it is specifically in the machining of the groove joints. The preparation of the V-groove for MIG and TIG joints is required for the AA5083-H11, as shown in Figure 12.

Considering that a milling process machines the V-groove and that the time needed to complete this groove can be calculated from Equation (2):

(2) $$T=T_{1}+T_{2}\;$$

where *T:* total time needed for the groove machining (min), *T*_1_*:* preparation (machine, tool, programming, and the like) time for the machining process is about 30–40 min, and *T*_2_*:* machining time (min).

**Figure 13:**
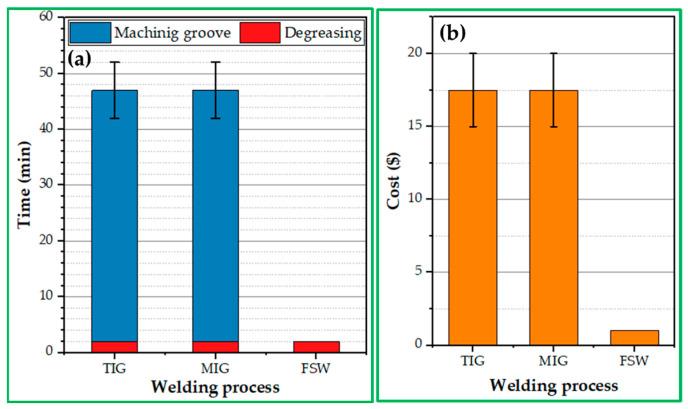
(**a**) the time and (**b**) the cost of preparation time needed to form the V-groove for the different welding techniques.

According to the calculation, the time required to prepare the MIG or TIG plates is 40–50 min, and the cost of machining them is around 15–20 USD. In the FSW process, material preparation costs are minimal—almost nonexistent. This technique does not require preheating or removing the protective oxide layer. Figure 13 illustrates the time and cost of the 5 mm thick and 1 m long AA5083-H111 preparation process for TIG, MIG, and FSW techniques.

**Figure 14:**
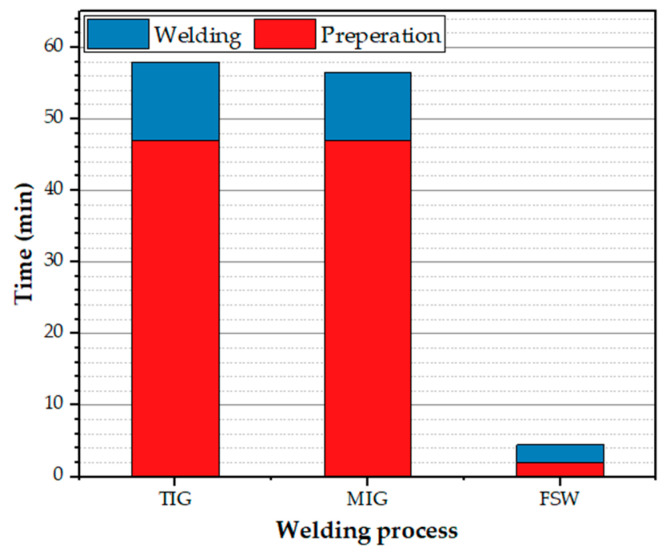
Time of production FSW, MIG, and TIG similar butt joints 5 mm thick and 1 m long AA5083-H111 alloy.

It can be mentioned that the TIG and MIG welding of 5 mm thick AA5083-H111 alloy is completed in three passes, whereas FSW welding is performed in one. The average welding speed of TIG and MIG is 290 mm/min, and based on the mechanical properties of the FSW joints, the recommended FSW travel speed is 400 mm/min. Therefore, the time needed to weld 1 m long of AA5083-H111 is approximately 11 min, 9.5 min for TIG and MIG welding processes, and 2.5 min for FSW. Figure 14 shows the time required for the procedures of MIG, TIG, and FSW processes to weld 1 m long and 5 mm thick AA5083-H1111.

#### 3.5.2. Cost Items

**Figure 15:**
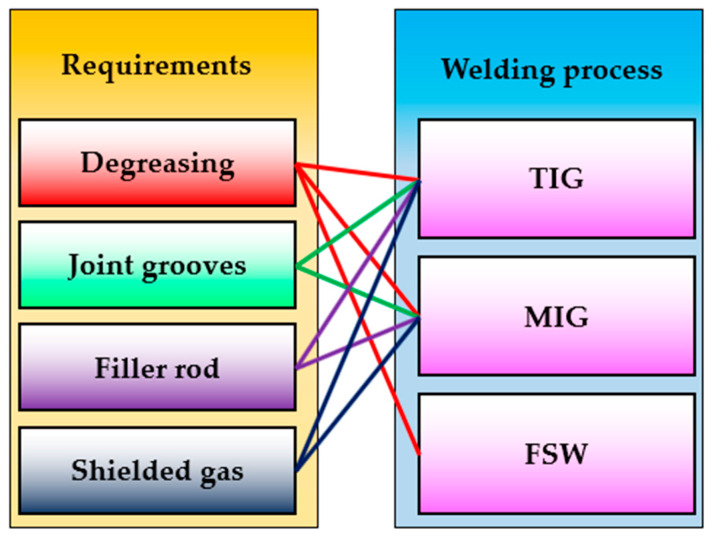
Comparison chart between the requirements of the TIG, MIG, and FSW processes to join a material.

The TIG technique may be conducted with or without additional material (filler rod). If the thickness of joint plates is less than 3 mm, a filler rod is not needed; if it is greater than 3 mm, a filler rod is required. This study requires the filler rod for the TIG and MIG processes, and the ER5356 filler rod is used as additional material. During FSW welding, no additional material is added; the FSW process is carried out without it. Shielded gas costs are another benefit of FSW over TIG and MIG. FSW welding does not need a shielding gas, unlike TIG and MIG. Based on the applied parameters listed in Table 4, the consumed shielding gas of argon during the TIG and MIG welding processes is approximately 209 and 180 L, respectively. Figure 15 shows the requirements for welding technologies for TIG, MIG, and FSW, which significantly affect the cost of the welding process.

## 4. Conclusions

The effect of three joining techniques, TIG and MIG as fusion welding processes and FSW as a solid-state welding process, on the quality and joint properties of AA5083-H111 aluminum alloy welded in similar butt joints was investigated. From the findings, we conclude the following:

1. The MIG joint processed at a welding current of 170 Amp, with a 19 L/min flow rate of pure argon and the FSW joints processed at a constant tool rotation speed of 400 rpm and two travel speeds of 100 and 400 mm/min using a cylindrical pin geometry revealed defect-free joints among all the welded AA5083-H111 butt joints.
2. The average hardness values of all the produced AA5083-H111 butt joints showed a notable enhancement over the hardness of AA5083-H111 BM by 25, 30, and 50% for the MIG, TIG, and FSW joints, respectively.
3. The FSW joints exhibit the highest efficiency, around 89%, compared with 70 and 83% for the MIG and TIG joints, respectively.
4. The highest joint tensile strength was obtained for the FSW joint produced at a travel speed of 400 mm/min and a 400 rpm rotation speed.
5. The FSW has an advantage over TIG and MIG welding techniques, including the time required to prepare specimens for the welding process, the welding time, and the number of welding passes. In contrast, TIG and MIG technologies require mechanical preparation, high heat input, and high occupational safety precautions.

## Figures and Tables

**Table 1:** Summarizes some recent works focused on comparison studies between the FSW process and fusion welding techniques.

No.	Welding Techniques	Joint Materials (Thickness)	Points of Comparison	Recommended Techniques	Ref.
1	FSW and TIG	AA7075 (4 mm)	Mechanical properties and microstructure	FSW	[33]
2	FSW and MIG	AA4007 (6 mm)	Mechanical properties	FSW	[34]
3	FSW and MIG	AA6061 T-6 (6 mm) and AA6082 T-6 (6 mm)	Mechanical properties and microstructure	MIG	[35]
4	FSW and TIG	AA5082 and AA7075 dissimilar joint (6 mm)	Mechanical properties	FSW	[36]

**Table 2:** The nominal composition of AA5083-H111 rolled plate.

Alloying Element	Mg	Zn	Si	Fe	Ti	Cu	Al
Wt. %	4.760	0.040	0.045	0.140	0.054	0.020	Balance

**Table 3:** The typical wire composition (according to the supplier of ESAB Company, Gothenburg, Sweden) is used in the MIG and TIG processes.

Element	Cr	Cu	Fe	Mg	Mn	Si	Zn	Al
Wt. %	0.12	0.01	0.13	4.90	0.13	0.05	0.01	Balance

**Table 4:** The applied MIG and TIG welding parameters to weld AA5083-H111 in butt joints.

Sample Code	Welding Process	Electrode or Filler Wire	Ampere (A)			Shielding Gas	Gas Flow Rate (L/min)	Welding Speed (mm/min)
			First Layer	Second Layer	Third Layer			
T1	TIG	R5356	Back 100 A	Root 135 A	Cap 100 A	Pure argon (99.99%)	19	240–300
T2			130	135	130			
M1	MIG	ER5356	Root 170 A	Cap 140 A	Back 140 A	Pure argon (99.99%)	19	290–350
M2			170 A	170 A	170 A			

**Table 5:** Visual inspection report of the AA5083-H111-butt-welded joint using the TIG, MIG, and FSW techniques.

Item	Sample No.	Welding Process	Joint Dimensions (L mm × W mm × T mm)	Evaluation	Remarks
1	T1	TIG	200 × 200 × 5	Accepted both sides	Reinforcement is about 2 mm The pass width is 12 mm
2	T2		200 × 200 × 5	Accepted both sides	Distortion in plates is not recommended Reinforcement is about 3 mm The path width is 11 mm
3	M1	MIG	200 × 200 × 5	Accepted both sides	Reinforcement is 3 mm The pass width is 12 mm
4	M2		200 × 200 × 5	Accepted both sides	Side (B) weld has light scratches at the start and should be removed Reinforcement is 1.5 mm The path width is 12.5 mm
5	F1	FSW	20 × 20 × 0.5	Accepted both sides	Little flash needs to be removed Keyhole to be refilling
6	F2		20 × 20 × 0.5	Accepted both sides	Little flash needs to be removed Keyhole to be refilling

**Table 6:** Summary of the RT test results of the AA5083-H111-butt-welded joint using the TIG, MIG, and FSW techniques.

Item	Sample No.	Welding Process	No. of Welding Passes	Evaluation	Remarks
1	T1	TIG	3	(0–5) LOF	-
				(5–18) LOP	
2	T2			(2–15) P	-
3	M1	MIG		(0 3) P	-
				(5–10) LOP	
4	M2		3	Accepted	Fully welded
5	F1	FSW	1	Accepted	Fully welded
6	F2			Accepted	Fully welded

## Data Availability

Not applicable.
